# The Impact of Hemodialysis on Humoral and Cellular Immunity in Patients with Renal Failure

**DOI:** 10.3390/jcm14186533

**Published:** 2025-09-17

**Authors:** Renad M. Alhamawi, Basmah Y. Shafea, Halah H. Bakhsh, Layal A. Fayraq, Samar T. Aloufi, Taraf F. Alharbi, Abdullah A. Alharbi, Abdulaziz A. Alharbi, Bashar F. Alanize, Abdulaziz M. Bakhsh, Emad S. Rajih, Ibrahim A. Sandokji, Waleed H. Mahallawi

**Affiliations:** 1 Clinical Laboratory Sciences Department, College of Applied Medical Sciences, Taibah University, Madinah 42353, Saudi Arabia; rhamawi@taibahu.edu.sa (R.M.A.); Basmash@outlook.sa (B.Y.S.); halah.h.bakhsh@gmail.com (H.H.B.); layalfayraq@outlook.com (L.A.F.); samaraloufi486@gmail.com (S.T.A.); taraf.alharbi@outlook.com (T.F.A.); abdullah.aa.alharb1@outlook.com (A.A.A.); abdulaziz.aali.alharbi@outlook.com (A.A.A.); bashar.alanezi@outlook.com (B.F.A.); 2 General and Specialized Surgery Department, College of Medicine, Taibah University, Madinah 42353, Saudi Arabia; bakhshaziz@gmail.com (A.M.B.); remad@taibahu.edu.sa (E.S.R.); 3 Department of Pediatrics, College of Medicine, Taibah University, Madinah 42353, Saudi Arabia; isandokji@gmail.com; 4 Health and Life Research Center, Taibah University, Madinah 42353, Saudi Arabia

**Keywords:** hemodialysis, renal failure, humoral immunity, cellular immunity

## Abstract

**Background**: End-stage renal disease (ESRD) is a growing global health concern, and hemodialysis (HD) remains the most common life-sustaining therapy for patients with advanced kidney failure. Both humoral and cellular immunity are impaired post hemodialysis, leading to immune system dysfunction. **Methods**: We utilized flow cytometry to quantify cell populations based on surface markers, including CD3 (total T lymphocytes), CD4 (helper T-cells), CD8 (cytotoxic T-cells), CD19 (B lymphocytes), and CD16/CD56 (natural killer (NK) cells). EDTA-blood samples were collected intravenously immediately before and after dialysis. **Results**: A consistent decline in CD3^+^ T lymphocytes was observed post hemodialysis. This reduction occurred across both male and female cohorts: *p* = 0.0342 and *p* = 0.0002, respectively. CD8^+^ cytotoxic T-cells decreased significantly post HD, *p* = 0.0003. Conversely, CD4^+^ helper T-cells exhibited a paradoxical increase, *p* = 0.0321. The divergent trends in CD4^+^ and CD8^+^ cells led to a statistically significant increase in the CD4/CD8 ratio post dialysis, *p* = 0.0005. Notably, stratification by gender uncovered that the post-HD changes in CD4^+^ and CD8^+^ T-cells were exclusive to female patients. Females demonstrated a pronounced increase in CD4^+^ cells and a sharper decline in CD8^+^ cells compared to males. CD19^+^ B lymphocytes showed a statistically significant decline post hemodialysis (*p* < 0.0001). While both genders exhibited reduced B-cell percentages, female patients experienced a more pronounced reduction than males. NK cells were severely depleted post dialysis in both male and female cohorts. **Conclusions**: Overall, the immune alterations observed in HD patients, including T-cell reduction, B-cell lymphopenia, and changes in NK cell populations, contribute to the increased risk of infections, malignancy, and cardiovascular disease in this population.

## 1. Introduction

End-stage renal disease (ESRD) is a growing global health concern, and hemodialysis (HD) remains the most common life-sustaining therapy for patients with advanced kidney failure. These patients showed impaired humoral immune response to vaccines such as the hepatitis B vaccine [1]. Therefore, further attention is required, such as a vaccination booster, the addition of adjuvants to the vaccine, or even an increase in the vaccine dose, to overcome this issue [2]. While HD is essential for removing toxins and maintaining fluid balance, it is also associated with a range of complications, including a high risk of infections, cardiovascular disease, and poor vaccine responses. These complications are closely linked to disturbances in the immune system, which are now recognized as a hallmark of chronic kidney disease and its treatment [3,4]. Indeed, impaired renal clearance in renal failure patients leads to the accumulation of uremic toxins, which contribute to immune dysfunction and promote systemic inflammation known as uremic hypercytokinemia [5].

Patients with ESRD have both immune senescence and chronic inflammation, which are associated with the so-called inflammaging syndrome [6]. The duality of persistent inflammation alongside weakened immune defenses contributes to the increased rates of infection, malignancy, and cardiovascular events observed in this population [7]. Both the innate and adaptive branches of the immune system are affected, with significant changes in the numbers and function of T-cells, B-cells [8], and natural killer (NK) cells [9]. B lymphocytes, responsible for antibody production, are also significantly affected. HD patients often have lower numbers of B-cells, especially switched memory B-cells, which are crucial for long-term immunity and effective vaccine responses [10]. This reduction in B-cell numbers and function mirrors changes seen in natural aging and is associated with increased susceptibility to infections and poor vaccine efficacy [11]. The mechanisms behind B-cell dysfunction are complex, involving uremic toxins, chronic inflammation, and impaired T-cell help [12].

NK cells, which play a crucial role in the innate immune response, also change in HD patients. Some studies report increased numbers of peripheral NK and NKT cells, while others found no significant change or even a reduction [13]. Functional changes in NK cells, including altered cytotoxic activity and cytokine production, have been linked to the chronic inflammatory state in HD patients and may contribute to the increased risk of cardiovascular disease and malignancy [14].

The mechanisms driving these immune changes are multifactorial. Uremic toxins, chronic inflammation, oxidative stress, and the bioincompatibility of dialysis membranes all contribute to immune dysfunction [15]. The HD procedure itself can cause acute changes in immune cell populations through complement activation, apoptosis, and shifts in cytokine profiles. The type and intensity of dialysis, as well as the use of therapies like erythropoietin, can also influence the extent of immune dysregulation [16].

Understanding how HD affects immune cell subsets is crucial for developing strategies to reduce complications and improve outcomes for patients with ESRD. Recent advances in technologies such as flow cytometry and single-cell sequencing have provided new insights into the complex changes occurring in the immune system during HD. Therefore, this study set out to assess the influence of hemodialysis on humoral and cellular immunity among renal failure patients in the Madinah region, Saudi Arabia, using the flow cytometry technique.

## 2. Material and Methods

### 2.1. Study Design and Data, and Sample Collection

This study included dialysis patients recruited from Hayat Organisation hemodialysis center, Madinah, Saudi Arabia. EDTA-blood samples were collected intravenously immediately before and after dialysis from these patients. Inclusion criteria: Adults (18–80 years) on maintenance HD for >3 months. Exclusion criteria: Active infection, immunosuppressive therapy, malignancy, or pregnancy. Written informed consent was obtained from all participants (IRB: 22-010). The study was conducted from February 2025 to April 2025. We declare that the investigations were conducted in accordance with the principles outlined in the Declaration of Helsinki (1975, revised in 2013).

The diagnosis of CKD in our study cohort was made in accordance with the KDIGO [17].

The criteria were as follows:

Presence of kidney damage for a duration of ≥3 months, indicated by one or more of the following markers:

Albuminuria (Albumin-to-Creatinine Ratio (ACR) ≥ 30 mg/g).

Electrolyte and other abnormalities due to tubular disorders.

Glomerular Filtration Rate (GFR) < 60 mL/min/1.73 m^2^ for ≥3 months.

As our study specifically focuses on patients with end-stage kidney disease (ESKD) on dialysis, all enrolled patients had progressed to CKD Stage 5 (GFR < 15 mL/min/1.73 m^2^) and required renal replacement therapy secondary to prerenal obstructive, and /or uropathy.

This study was conducted at a single center, with a total of 184 eligible patients identified during the study period. Of these, 28 patients met the inclusion criteria and agreed to participate. Given the limited nature of the population, we applied margin-of-error estimation for proportions with limited population correction. Assuming maximum variability (*p* = 0.5), a sample of 28 out of 184 yields an estimated margin of error of approximately ±12% at the 95% confidence level. All the patients were prescribed a three-times-a-week schedule. Additionally, all patients were taking Erythropoietin (60 mg).

Fresenius Medical Care AG & Co. KgaA, model 5008s dialyzer HD (Bad Homburg, Germany), but not Hemodiafiltration (HDF), was used for all patients. Data on the specific biocompatibility or composition of the dialysis membranes used for each patient (e.g., polysulfone, polyethersulfone, cellulose triacetate) were not consistently available in the medical records and were therefore not analyzed as a variable in this study. Additionally, guidelines recommended a Kt/V of 1.2 as the minimum dose for thrice-weekly HD [18]. Our cohort Kt/V dose ranged from 1.2 to 1.6, with an average of 1.3. Hence, if Kt/V was >1.4, the treatment was considered very effective, while if Kt/V was below 1.2 (in HD), this suggested longer sessions, a higher blood flow rate, and better vascular access. Although Kt/V is a useful marker of dialysis adequacy, clinical studies have shown that Kt/V is not often the sole or most consistent determinant of dialysis outcomes [19].

Also, sociodemographic data (age, gender, and cause of renal failure) were collected from patients’ files. Blood samples were collected before and immediately after the HD procedure. All participants signed a consent form prior to data or sample collection.

### 2.2. Flow Cytometry

Peripheral blood mononuclear and polymorphonuclear cells were isolated from EDTA-blood samples collected from each patient immediately (before and after dialysis session). Briefly, 1 mL of red blood cell lysing buffer (Qiagen, Germantown, MD, USA) was added to 1 mL of the whole blood sample for 10–15 min. Then, the lysed blood samples were washed with phosphate-buffered saline (PBS) and centrifuged for 5 min at 1500 rpm to obtain the cell pellets for flow cytometry staining. All samples were stained with BD Multitest™ 6-Colour TBNK (BD Biosciences, San Jose, CA, USA) for the identification of T-cells, CD4^+^ T-cells, CD8^+^ T-cells, B-cells and natural killer (NK) cells for 30 min at 4 °C. The reagent was 6 surface markers with different fluorochromes, including anti-CD45 (PerCPCy5.5), anti-CD3 (FITC), anti-CD4 (PE-CY7), anti-CD8 (APC-CY7), anti-CD19 (APC), and anti-CD16/CD56 (PE). Then, cells were washed and resuspended with PBS and run on the Attune Flow Cytometer (Thermo Fisher, Waltham, MA, USA). All flow cytometric data were analyzed using FlowJo version 10 (BD Biosciences, San Jose, CA, USA).

### 2.3. Statistical Analysis

All tests and graphical representations in this study were conducted using GraphPad Prism version 10 (San Diego, CA, USA). The paired non-parametric (Wilcoxon signed-rank test) was used to compare the frequency of immune cells of dialysis patients before and after dialysis. A 95% confidence level was applied to determine the significance of the data set. *p*-values less than 0.05 were considered statistically significant. * < 0.05, ** < 0.01, *** < 0.001, **** < 0.0001.

## 3. Results

### 3.1. Sample Characteristics

The main aim of this study was to evaluate the impact of hemodialysis on the proportion of immune cells, including T lymphocytes, B lymphocytes, and NK cells, among renal failure patients. A total of 28 renal failure patients on hemodialysis participated in this study. The majority of participants were in the 41 to 50 years old and 51 to 60 years old age groups (35.7%, *n* = 10, 32.1% *n* = 9, respectively). About 42.9% of participants were male, while 57.1% were female. The leading cause of renal failure among participants was hypertension, affecting 19 patients (67.8%). Moreover, eight participants were suffering from both diabetes and hypertension, leading to renal failure, while only one patient had renal hypoplasia.

Anemia was prevalent, with 50.0% of patients having mild anemia (mean hemoglobin 11 ± 0.9 mg/dL), 21.4% moderate anemia (mean 9 ± 0.55 mg/dL), and 28.6% within the normal range (mean 13 ± 0.64 mg/dL); no cases of severe anemia were reported. Elevated ferritin levels were observed in 92.8% of the cohort (mean 723.2 ± 333.7 mg/dL), while low ferritin and normal levels were each seen in only 3.6% of patients.

Hypocalcemia was identified in 60.7% of patients (mean calcium 7.7 ± 0.55 mg/dL), whereas 39.3% had normal calcium levels; no cases of hypercalcemia were found. Phosphorus levels were normal in 57.2% of individuals, with 35.7% showing hyperphosphatemia (mean 6.2 ± 0.39 mg/dL) and 7.1% hypophosphatemia (mean 1.35 ± 0.07 mg/dL). Parathyroid hormone (PTH) abnormalities were common, with 53.6% of patients exhibiting hyperparathyroidism (mean PTH 1210.2 ± 753.2 pg/mL), 39.3% having normal levels, and 7.1% presenting with hypoparathyroidism.

Albumin levels were within the normal range for the majority of patients (96.4%, mean 3.92 ± 0.32 g/dL), one patient (3.6%) had elevated albumin (7.1 g/dL), and no cases of hypoalbuminemia were observed. The characteristics of the participants are detailed in Table 1.

### 3.2. The Influence of Hemodialysis Sessions on Different Immune Subsets Based on Gender and Age

**Figure 1:**
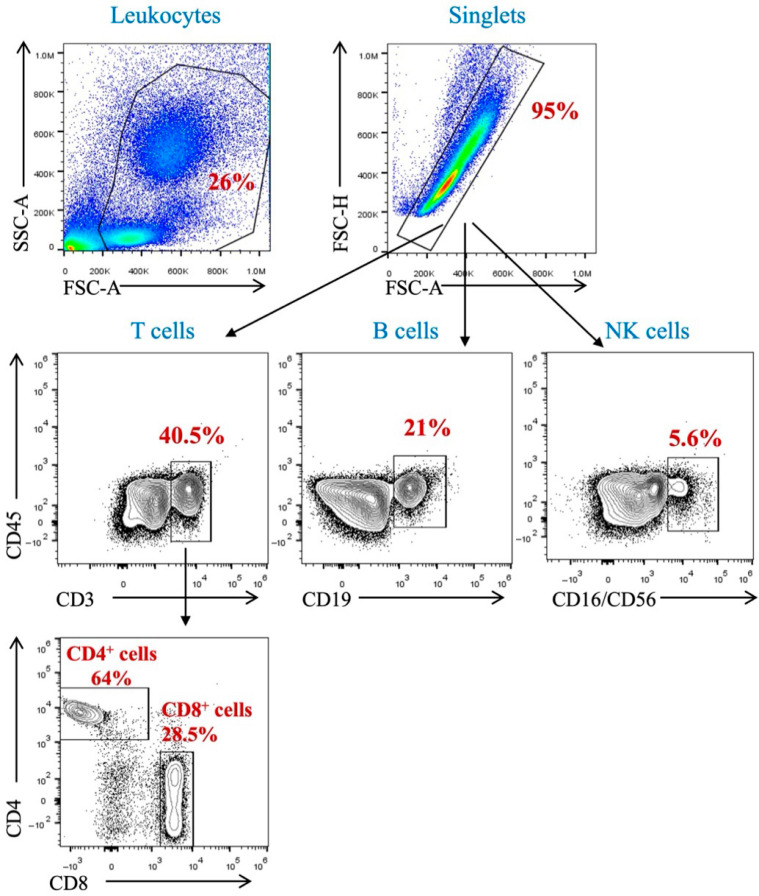
Gating strategy for the identification of immune population (T-cells, B-cells, and NK cells) in peripheral blood.

The impact of hemodialysis sessions on peripheral immune cells such as T-cells, B-cells, and natural killer (NK) cells was assessed among renal failure patients. The percentage of peripheral immune cells detected in these patients before and after dialysis was analyzed using flow cytometric technique. A gating strategy was set up to identify immune subsets in the participants’ peripheral blood samples. Using the surface markers CD45, CD3, CD4, CD8, CD19, CD16, and CD56, we identified T-cell subpopulations, B-cells, and NK cells among peripheral blood mononuclear cells (PBMCs) and polynuclear cells. Leukocytes were identified due to their forward- and side-scatter profiles. Single cells were identified by forward Height and area. T-cells were identified as being CD45- and CD3-positive cells; then, we applied gating to identify CD4 and CD8 T-cells. B-cells were CD19- and CD45-positive cells, while NK cells were CD16/CD56- and CD45-positive cells (Figure 1).

**Figure 2:**
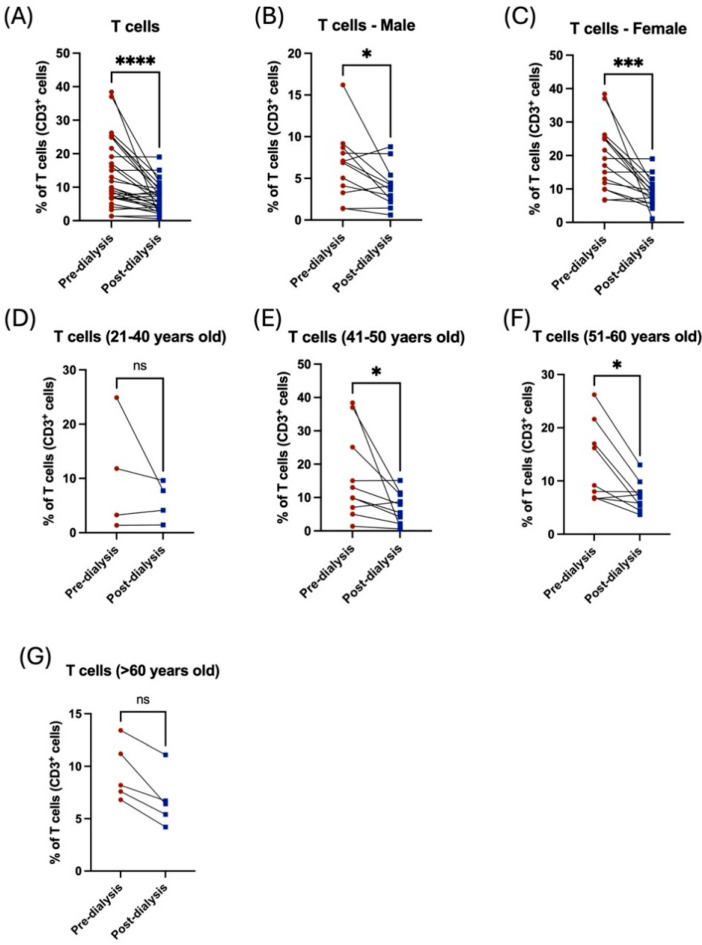
The effect of hemodialysis on CD3^+^ T lymphocytes based on gender and age stratification. (**A**) Percentage of the total peripheral T-cells of patients before and after dialysis. (**B**) Percentage of the total peripheral T-cells before and after dialysis among male patients. (**C**) Percentage of the total peripheral T-cells before and after dialysis among female patients. (**D**) Percentage of the total peripheral T-cells before and after dialysis among 21–40 year-old patients. (**E**) Percentage of the total peripheral T-cells before and after dialysis among 41–50 year-old patients. (**F**) Percentage of the total peripheral T-cells before and after dialysis among 51–60 year-old patients. (**G**) Percentage of the total peripheral T-cells before and after dialysis among >60 year-old patients. * < 0.05, *** < 0.001, **** < 0.0001, ns = non-significant.

Interestingly, there was a noticeable reduction in peripheral CD3^+^ T-cells after dialysis (Figure 2A). The proportion of total T lymphocytes was significantly affected by dialysis among both male and female patients (Figure 2B,C, Table 2). Middle-aged patients (41–60 years old) showed a significant reduction in total T-cells post dialysis session (Figure 2E,F, Table 3). However, the younger cohort, those younger than 40 years, and those older than 60 years, showed a noticeable decrease in T-cell population, but this was non-significant (Figure 2D,G, Table 3).

**Figure 3:**
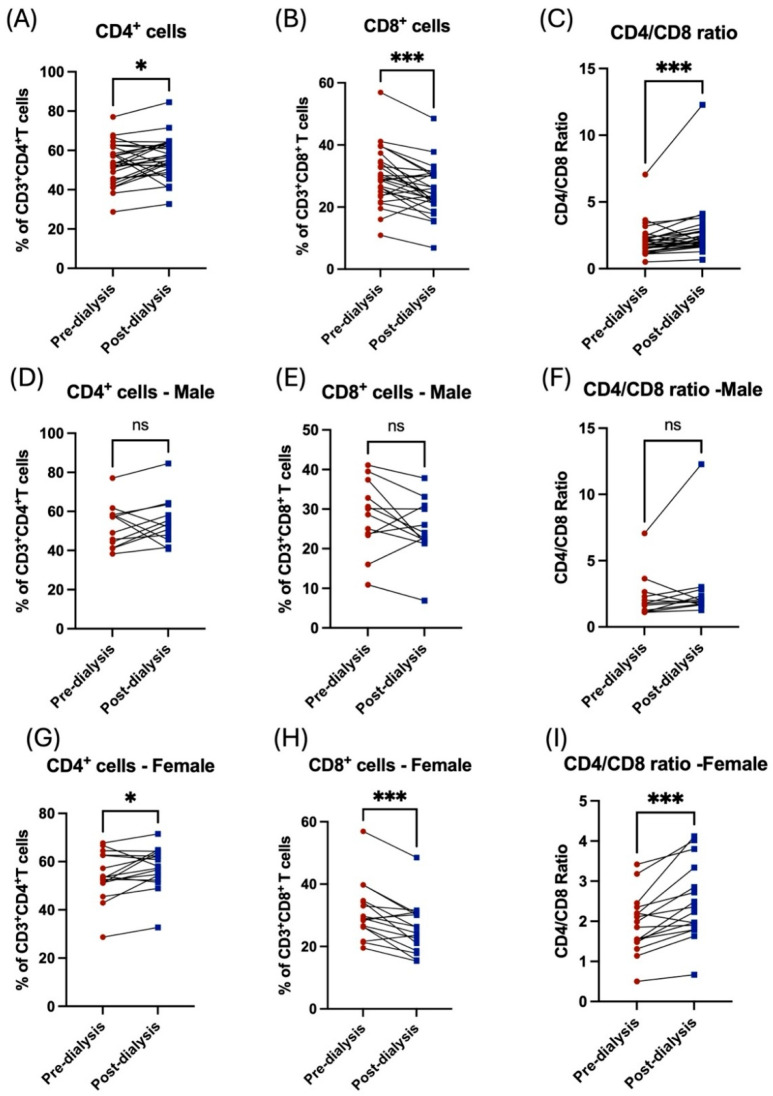
The impact of hemodialysis on T lymphocyte subpopulation (CD4^+^ and CD8^+^ T-cells) based on gender stratification. (**A**) Percentage of total CD4^+^ T-cells before and after dialysis among participants. (**B**) Percentage of total CD8^+^ T-cells before and after dialysis among participants. (**C**) Ratio of CD4/CD8 before and after dialysis among participants. (**D**) Percentage of total CD4^+^ T-cells before and after dialysis among male patients. (**E**) Percentage of total CD8^+^ T-cells before and after dialysis among male patients. (**F**) Ratio of CD4/CD8 before and after dialysis among male patients. (**G**) Percentage of total CD4^+^ T-cells before and after dialysis among female patients. (**H**) Percentage of total CD8^+^ T-cells before and after dialysis among female patients. (**I**) Ratio of CD4/CD8 before and after dialysis among female patients. * < 0.05, *** < 0.001, ns = non-significant.

**Figure 4:**
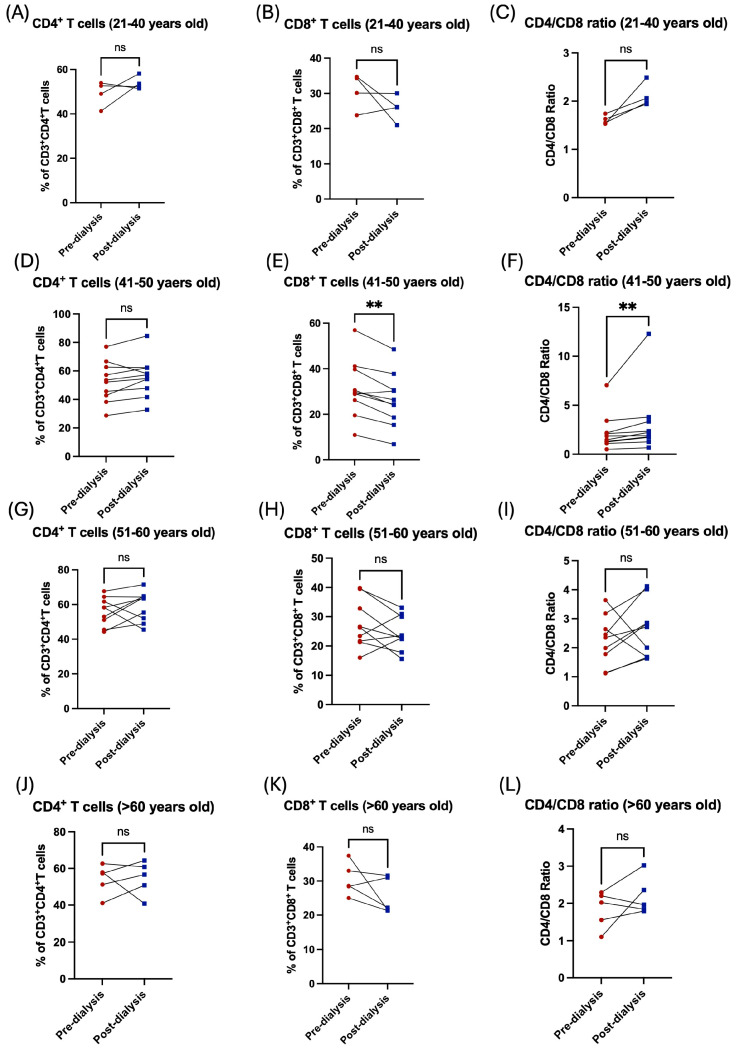
The impact of hemodialysis on T lymphocyte subpopulation (CD4^+^ and CD8^+^ T-cells) based on age stratification. (**A**) Percentage of total CD4^+^ T-cells before and after dialysis among 21–40 year-old patients. (**B**) Percentage of total CD8^+^ T-cells before and after dialysis among 21–40 year-old patients. (**C**) Ratio of CD4/CD8 before and after dialysis among 21–40 year-old patients. (**D**) Percentage of total CD4^+^ T-cells before and after dialysis among 41–50 year-old patients. (**E**) Percentage of total CD8^+^ T-cells before and after dialysis among 41–50 year-old patients. (**F**) Ratio of CD4/CD8 before and after dialysis among 41–50 year-old patients. (**G**) Percentage of total CD4^+^ T-cells before and after dialysis among 51–60 year-old patients. (**H**) Percentage of total CD8^+^ T-cells before and after dialysis among 51–60 year-old patients. (**I**) Ratio of CD4/CD8 before and after dialysis among 51–60 year-old patients. (**J**) Percentage of total CD4^+^ T-cells before and after dialysis among >60 years old patients. (**K**) Percentage of total CD8^+^ T-cells before and after dialysis among >60 year-old patients. (**L**) Ratio of CD4/CD8 before and after dialysis among >60 year-old patients. ** < 0.01, ns = non-significant.

Similarly, the influence of the hemodialysis session on these patients was shown through the decrease in the subpopulation of T-cells. For CD8^+^ cytotoxic T, however, this was not the case among the CD4^+^ helper population, which was shown to be increased post dialysis (Figure 3A,B). Additionally, the CD4/CD8 ratio was affected and showed a significant increase post dialysis (Figure 3C). Stratification of the patients based on their gender showed the influence of hemodialysis on T-cell subpopulation; CD4^+^ and CD8^+^ T-cells were detected in female patients only (Figure 3D–I, Table 2). Only patients aged between 41 and 50 years showed a significant decrease in CD8^+^ T-cells and a consequent an increase in CD/CD8 ratio (Figure 4E,F, Table 3). All other age groups showed non-significant differences post dialysis session (Figure 4A–L, Table 3).

**Figure 5:**
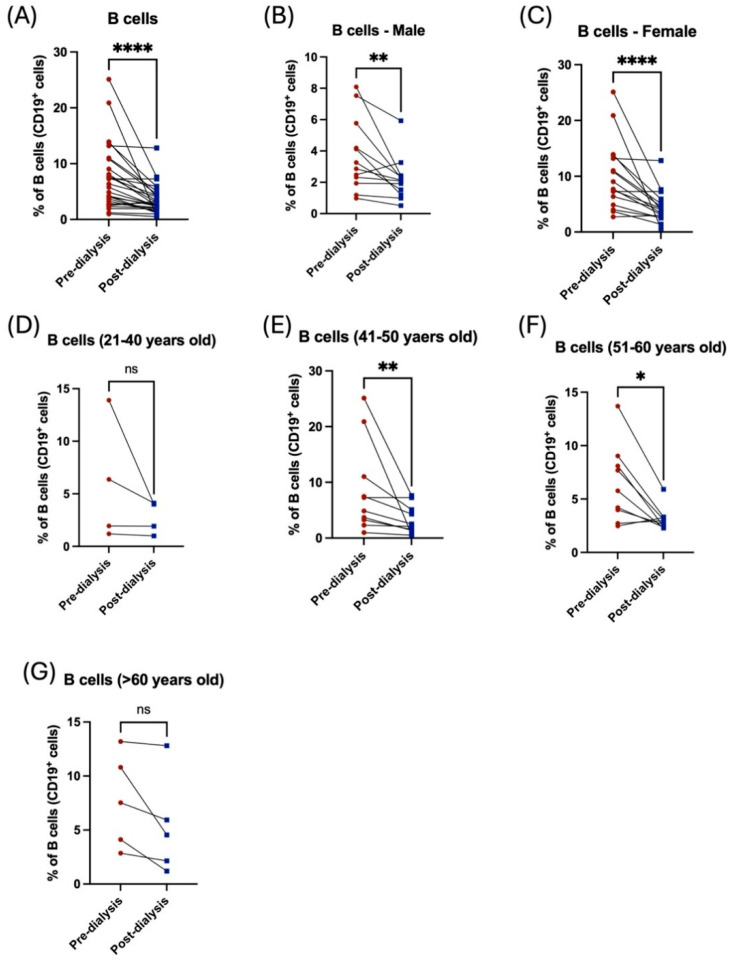
The influence of hemodialysis on B lymphocytes among male and female patients and different age groups. (**A**) Percentage of the total peripheral B-cells before and after dialysis. (**B**) Percentage of the total peripheral B-cells before and after dialysis among male patients. (**C**) Percentage of the total peripheral B-cells before and after dialysis among female patients. (**D**) Percentage of the total peripheral B-cells before and after dialysis among 21–40 year-old patients. (**E**) Percentage of the total peripheral B-cells before and after dialysis among 41–50 year-old patients. (**F**) Percentage of the total peripheral B-cells before and after dialysis among 51–60 year-old patients. (**G**) Percentage of the total peripheral B-cells before and after dialysis among >60 year-old patients. * < 0.05, ** < 0.01, **** < 0.0001, ns = non-significant.

Analysis of CD19^+^ B lymphocytes exhibited a statistically significant decrease in the proportion of B lymphocytes post hemodialysis (Figure 5A). Noticeably, both male and female patients showed a reduction in the percentage of such cells; however, female patients were more affected by dialysis than male patients (Figure 5B,C, Table 2). Patients aged between 41 and 60 years showed a significant reduction in the percentage of B lymphocytes (Figure 5E,F, Table 3), while younger and older patients showed a non-significant decrease in B-cells (Figure 5D,G, Table 3).

**Figure 6:**
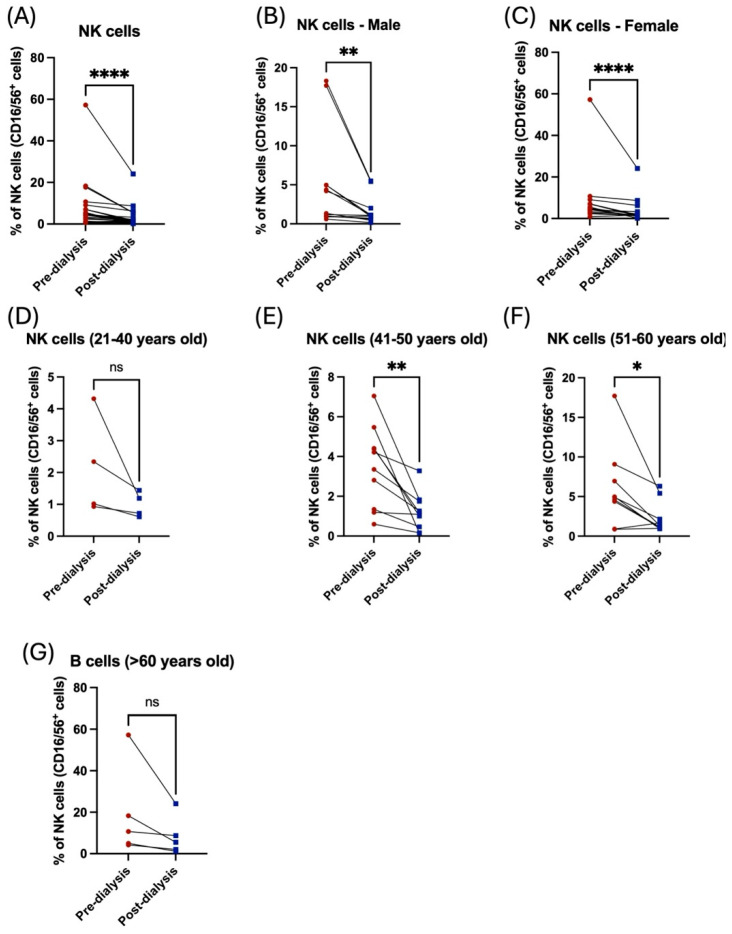
The effect of hemodialysis on NK cells among male and female patients and different age groups. (**A**) Percentage of the total peripheral NK cells before and after dialysis. (**B**) Percentage of the total peripheral NK cells before and after dialysis among male patients. (**C**) Percentage of the total peripheral NK cells before and after dialysis among female patients. (**D**) Percentage of the total peripheral NK cells before and after dialysis among 21–40 year-old patients. (**E**) Percentage of the total peripheral NK cells before and after dialysis among 41–50 year-old patients. (**F**) Percentage of the total peripheral NK cells before and after dialysis among 51–60 year-old patients. (**G**) Percentage of the total peripheral NK cells before and after dialysis among >60 year-old patients. * < 0.05, ** < 0.01, **** < 0.0001, ns = non-significant.

Likewise, NK cells showed a severe reduction post dialysis among both male and female patients (Figure 6C, Table 2). Only middle-aged patients had a statistically significant decrease in NK cell population post dialysis (Figure 6D–G, Table 3). Noticeably, the frequency of NK cells was higher in older patients compared to the young cohort (Table 3). Taken together, these data demonstrated the influence of hemodialysis on immune cells among renal failure patients, highlighting the importance of evaluating the function of the immune system to prevent the occurrence of inflammatory diseases among these patients.

Peripheral blood mononuclear and polymorphonuclear cells were isolated from EDTA-blood samples collected from each participant. Cells were stained with anti-CD45, anti-CD3, anti-CD4, anti-CD8, anti-CD19, and anti-CD16/CD56.

## 4. Discussion

The critical role of chronic systemic inflammation in End-Stage Renal Disease (ESRD) and dialysis patients is well-established as a central driver of the profound immune dysfunction observed in this population. This persistent, low-grade inflammatory state arises from a multifactorial etiology, including the accumulation of uremic toxins (particularly gut-derived protein-bound solutes like indoxyl sulfate and p-cresyl sulfate), recurrent immune activation from bio-incompatible dialysis materials, and increased gut permeability leading to endotoxin exposure [20]. This inflammatory milieu directly contributes to immune dysfunction by promoting a state of simultaneous immune activation and exhaustion. It drives premature immunosenescence, characterized by T-cell exhaustion [21], impaired neutrophil and natural killer (NK) cell function, and reduced antigen-presenting cell efficacy, which collectively heighten susceptibility to infections, diminish vaccine responsiveness, and increase the risk of virus-associated malignancies [22]. Furthermore, this inflammation is a key pathological link between immune dysfunction and the vastly elevated cardiovascular risk in ESRD, as pro-inflammatory cytokines like IL-6 and TNF-α promote endothelial dysfunction, atherosclerosis, and vascular calcification [23]. Consequently, chronic systemic inflammation is not merely a biomarker but a key pathogenic force shaping the uremic immune phenotype and its associated clinical consequences [24].

The study underscores hemodialysis as a potent modulator of peripheral immunity, with distinct effects on adaptive (T- and B-cells) and innate (NK cells) compartments. Interestingly, our result showed an elevation in CD4/CD8 ratios and gender-specific subset changes. Compromised innate immunity may heighten vulnerability to viral infections or malignancies. These changes in immune cells in hemodialysis patients may be a potential mechanism. For example, contact with bioincompatible dialysis membranes, fluid removal, or electrolyte shifts may trigger leukocyte activation, apoptosis, or margination [25]. Additionally, gender differences, possibly due to estrogen’s role in enhancing CD4^+^ T-cell activity or modulating B-cell survival, warrant further exploration [26].

Hemodialysis (HD) has a profound impact on the immune system, particularly on T-cells, B-cells, and natural killer (NK) cells. HD is associated with a chronic inflammatory state and a significant reshaping of the acquired immune system, particularly in T-cell populations [27]. Previous studies showed that HD leads to an impairment of CD3^+^ T-cells, accompanied by an increase in CD4^+^ and CD8^+^ T-cell populations that produce pro-inflammatory cytokines, such as IL-17 and IFN-γ, indicating a shift toward a pro-inflammatory immune profile [28].

At the molecular level, HD suppresses the expression of T-cell receptor (TCR) genes in CD4^+^ T-cell subsets and inhibits downstream signaling pathways, including the PI3K-Akt-mTOR and NF-κB pathways, which are crucial for T-cell function [29]. T-cells, which are central to adaptive immunity, show both quantitative and qualitative changes in HD patients. Studies have reported reduced overall T-cell counts, altered CD4^+^/CD8^+^ ratios, and an increase in T-cells with a senescent or exhausted phenotype. These changes are not only a result of uremia but are also directly influenced by the HD procedure itself, which can trigger apoptosis and shift the balance of T-cell subsets [30]. Therefore, the increased proportion of CD4^+^ cells post dialysis, which led to an imbalance in the CD4^+^/CD8^+^ T-cell ratio, could be due to a reduction in Th2 and regulatory T-cells (Treg), and an altered interaction with B lymphocytes mediated by CD40/CD40L [31]. The observed decline in total CD3^+^ T-cells post HD aligns with a study linking HD to Fas/FasL-mediated apoptosis and mechanical shear stress during extracorporeal circulation [32].

Another explanation for this alteration suggests that these mechanisms are exacerbated by bioincompatible dialyzer membranes, which activate caspase-3 and trigger mitochondrial depolarization in lymphocytes [33]. The paradoxical increase in CD4^+^ helper T-cells, however, contrasts with the CKD-associated CD4^+^ lymphopenia, which was proposed to be transient margination of memory CD4^+^ T-cells into lymphoid tissues during HD [25].

The CD4^+^ T-cell count, as a percentage of lymphocytes and as an absolute number, increased immediately following hemodialysis in both participants with HIV and controls. These findings contradict previously published data, which suggest that absolute CD4^+^ T-cell counts decline immediately post dialysis [34]. The apparent increase in CD4^+^ T-cells may reflect the loss of CD8^+^ T-cells and concomitant hemoconcentration. The CD4^+^ T-cells may also have been recruited from other areas, such as the solid lymphoid tissue. The effector functions of these cells are uncertain. Importantly, no decrease was reported in CD4^+^ T-cells in the immediate post-dialysis period. No patients developed a CD3^+^ T-cell lymphopenia or a decreased CD8^+^ T-cell count before dialysis, although patients with HIV had significantly lower CD4^+^ T-cell counts before dialysis than controls [34].

The gender-specific CD4^+^/CD8^+^ shifts in females, marked by a pronounced CD4^+^ rise and CD8^+^ decline, suggest hormonal modulation of immune responses. Furthermore, studies have shown that estrogen enhances Th2 polarization and CD4^+^ survival, while testosterone suppresses CD8^+^ T-cell activation [35,36]. These findings align with murine models where ERα knockout blunted post-inflammatory CD4^+^ recovery, underscoring estrogen’s protective role in females [37]. Conversely, the lack of CD8^+^ decline in males may reflect testosterone’s immunosuppressive effects, buffering cytotoxic T-cell loss [38]. It is worth noting that, to reach a conclusive judgment on the effect of sex hormones on alterations in these immune cells, a larger number of patients and more multicenter data should be studied.

Memory B-cell depletion is a hallmark of CKD [39]. The CD19^+^ B-cell decline post HD corroborates reports of TLR4/NF-κB dysregulation and oxidative stress-induced apoptosis. Uremic toxins like IS inhibit B-cell receptor signaling, impairing antibody diversification, while HD-induced ROS generation further depletes memory B-cells [40,41]. Females exhibited a more pronounced B-cell loss, likely due to estrogen’s enhancement of B-cell activation via TLR7/9 pathways, rendering them susceptible to HD-induced apoptosis [42].

Additionally, we observed a significant decrease in the proportion of NK cells, which may contribute to a permissive environment for viral reactivation. Indeed, previous studies have shown that patients undergoing hemodialysis exhibit higher Varicella Zoster Virus (VZV) seroprevalence and elevated antibody levels compared to healthy controls [43], which is potentially linked to a reduction in the NK cell population. The severe NK cell depletion post HD aligns with the results of [44], who identified mitochondrial oxidative stress and NKG2A upregulation as key drivers of NK cell exhaustion. NK cells are critical for controlling CMV reactivation, which affects 30–50% of HD patients and correlates with cardiovascular morbidity. Biocompatible dialyzers (e.g., vitamin E-coated) mitigate NK cell loss by scavenging ROS and reducing mitochondrial damage and advocating for their broader clinical adoption [45]. Although the current study did not account for potential variations in hemodialysis techniques and, crucially, the biocompatibility of dialysis membranes, it is well-established that bioincompatible membranes (e.g., unsubstituted cellulose) can potentially activate the complement system and leukocytes, leading to an increase in the release of pro-inflammatory cytokines such as IL-1, IL-6, and TNF-α [46]. Conversely, more biocompatible synthetic membranes (e.g., polysulfone and polyethersulfone) are associated with attenuated inflammatory responses and potentially better preservation of nutritional status and immune cell function [31]. Thus, future prospective studies should meticulously document and control for dialysis membrane composition to isolate its specific contribution to immune modulation in HD patients.

The interplay between end-stage renal disease (ESRD) and immune dysfunction is complex, involving both the uremic milieu and the repetitive bio-incompatible challenges of hemodialysis (HD) itself. Our study provides a detailed characterization of the profound alterations in both the adaptive and innate immune systems in a cohort of chronic HD patients. We demonstrate that hemodialysis acts as a potent modulator of peripheral immunity, with distinct and contrasting effects on adaptive (T- and B-cells) and innate (Natural Killer (NK) cells) compartments. These findings are consistent with a growing body of literature documenting a state of chronic inflammation and immune exhaustion in this vulnerable population [47].

Impact on Adaptive Immunity—T-cell Exhaustion and Dysregulation: Our observation of a significantly reduced CD4^+^/CD8^+^ T-cell ratio, driven primarily by the relative expansion of CD8^+^ T-cells, aligns with previous studies investigating the immunological impact of single HD sessions. The research indicates that the HD procedure itself can contribute to T-cell lymphopenia, partly through the induction of apoptosis in CD8^+^ T-cells. Furthermore, the accumulation of differentiated, potentially senescent T-cells and reduction in naïve T-cells are well-documented phenomena in ESRD, resulting from both the loss of renal function and repeated immune activation during dialysis. This shift towards a terminal effector phenotype is indicative of immune exhaustion, a state characterized by impaired proliferative capacity and reduced effector function, which may explain the inadequate response to novel antigens and vaccinations observed in HD patients [48].

Our data, showing elevated levels of pro-inflammatory cytokines, corroborate findings that HD with bio-incompatible materials triggers the release of IL-1, IL-6, and TNF-α from monocytes and macrophages. This persistent inflammatory pressure promotes continuous T-cell activation, as evidenced by the increased expression of activation markers like HLA-DR, which we and others have observed [49].

B-cell Compartment—Imbalance and Functional Impairment: Our analysis revealed a significant imbalance in B-cell subpopulations. We found a higher percentage of memory B-cells and a concomitant reduction in immature B-cells in our pre-dialysis patients compared to healthy controls, a profile that was somewhat modulated in patients on maintenance HD. This finding is supported by a study specifically focused on B-cell-associated immune profiles in ESRD, which also reported an increase in memory B-cells and a decrease in immature B-cells in pre-dialysis patients, suggesting an activated yet dysregulated immune state due to uremia [50].

Compromised Innate Immunity—NK Cell Dysfunction: A critical finding of our study is the significant depletion and functional impairment of NK cells. NK cells are a vital first line of defense against viral infections and malignant cells. Most studies have confirmed that NK cell cytotoxicity is decreased in HD patients. This dysfunction is multifactorial, attributed to the uremic environment itself, which induces oxidative stress and downregulates the expression of pivotal activating receptors like NKG2D on NK cells [51]. Furthermore, the biocompatibility of the dialysis membrane plays a crucial role; bioincompatible membranes (e.g., cuprophane) have been shown to elicit a higher proportion of NK cells but lead to a drastic decrease in their cytotoxicity, while more biocompatible synthetic membranes cause less-pronounced dysfunction. Theoretically, this reduced NK cell number and function directly increases susceptibility to viral infections (e.g., CMV, HBV) and cancer development, which are highly prevalent and impactful in the ESKD population [52].

Cardiovascular Disease: The persistent inflammatory state, driven by chronic immune activation and pro-inflammatory cytokine release (e.g., IL-6, TNF-α), is a well-established, powerful driver of atherosclerosis and cardiovascular mortality in dialysis patients [24]. Activated immune cells infiltrate vascular walls, promote plaque instability, and contribute to endothelial dysfunction. Recent studies have demonstrated that composite inflammatory indices, such as the Systemic Immune–Inflammation Index (SII) and Systemic Inflammation Response Index (SIRI) [27], which integrate neutrophil, lymphocyte, and platelet counts, are strong independent predictors of all-cause and cardiovascular mortality in the HD population.

Infections and Malignancies: The functional impairment of NK cells, combined with T-cell exhaustion and B-cell dysregulation, creates an immunoparalysis state that heightens susceptibility to bacterial and viral infections. Infections are the second leading cause of death in HD patients [53]. Furthermore, impaired immune surveillance due to NK cell dysfunction and inadequate T-cell response is a key facilitator of cancer development and progression, explaining the elevated risk of virus-associated and other malignancies in this population [54].

Understanding the impact of hemodialysis on the immune system is crucial to preventing the occurrence of infectious and inflammatory diseases among these patients. However, this study has several limitations that should be taken into consideration. First, the relatively small sample size may restrict the generalizability of the findings and limit the statistical power to detect more nuanced effects. Second, while the observed alterations in the frequencies of different immune subsets following dialysis are notable, it remains unclear whether these disturbances are persistent or transient. Dialysis is known to cause short-term fluctuations in leukocyte distribution due to fluid shifts and cellular reallocation, which may not reflect long-term immunological changes. Therefore, longitudinal studies with larger, more diverse cohorts are needed to clarify the duration and clinical significance of these immune alterations in dialysis patients. While this level of precision is acceptable for an exploratory study, we acknowledge the limitation and recommend future studies with larger samples to confirm and generalize our findings. Lastly, performing additional laboratory tests, including biochemical markers such as urea, creatinine, uric acid, inflammatory markers, and electrolytes, and correlating the findings of immune cell alterations with the patient’s history of recent infection or vaccination would help make the study clinically useful for decision-makers. Therefore, our findings could provide valuable information to those responsible for caring for HD patients when considering these fundamental immune cell alterations. Additionally, taking these alterations into account when measuring the cells at a certain time point post dialysis could help to monitor the immune status, to avoid any misdiagnosis and improve the treatment plan.

## 5. Conclusions

In conclusion, hemodialysis is a potent modulator of peripheral immunity, with distinct effects on adaptive (T- and B-cells) and innate (NK cells) compartments. Compromised innate immunity may heighten vulnerability to viral infections or malignancies. These findings support the use of gender-stratified immune-monitoring and therapeutic strategies. Future research should prioritize longitudinal studies to distinguish acute HD effects from cumulative immune exhaustion, alongside omics profiling (single-cell RNA sequencing) to identify HD-specific immune signatures. To the best of our knowledge, our study is the first to have been conducted in Saudi Arabia. Thus, further investigations should be conducted, including different rejoin experiments, to gain a comprehensive understanding of the influence of HD on additional immune cells.

## Figures and Tables

**Table 1:** Sample characteristics (*n* = 28).

	*n*	%	Mean ± SD
Age			
21–40 years old	4	14.3	32 ± 5.35
41–50 years old	10	35.7	46 ± 2.65
51–60 years old	9	32.1	56 ± 3.4
>60 years old	5	17.9	73 ± 10
Gender			
Male	12	42.9	-
Female	16	57.1	-
Renal failure causes			
Renal hypoplasia	1	3.6	-
Hypertension	19	67.8	-
Hypertension and Diabetes Mellitus	8	28.6	-
Anemic Status (Hemoglobin mg/dL)			
Normal	8	28.6	13 ± 0.64
Mild	14	50.0	11 ± 0.9
Moderate	6	21.4	9 ± 0.55
Severe	0	0	0
Ferritin mg/dL			
Normal	1	3.6	298
Low	1	3.6	17.4
High	26	92.8	723.2 ± 333.7
Calcium mg/dL			
Normal	11	39.3	9 ± 0.26
Hypocalcemia	17	60.7	7.7 ± 0.55
Hypercalcemia	0	0	0
Phosphorus mg/d			
Normal	16	57.2	4.2 ± 0.8
Hypophosphatemia	2	7.1	1.35 ± 0.07
Hyperphosphatemia	10	35.7	6.2 ± 0.39
Parathyroid hormone pg/mL			
Normal	11	39.3	349.2 ± 114.4
Hypoparathyroidism	2	7.1	88.45 ± 0.78
Hyperparathyroidism	15	53.6	1210.2 ± 753.2
Albumin g/dL			
Normal	27	96.4	3.92 ± 0.32
Hypoalbuminemia	0	0	0
Hyperalbuminemia	1	3.6	7.1

**Table 2:** The effect of the hemodialysis session on different subsets of immune cells based on gender stratification.

Immune Cells	Males			Females		
	Pre-Dialysis	Post-Dialysis	*p* Value	Pre-Dialysis	Post-Dialysis	*p* Value
T cells	6.5 ± 4	4 ± 2.4	0.034	19.24 ± 9.8	9 ± 4.3	0.0002
CD4^+^ T-cells	52.5 ± 11.2	54.8 ± 12.02	0.531	54.1 ± 9.9	57.3 ± 8.8	0.039
CD8^+^ T-cells	28.3 ± 9.1	24.9 ± 7.8	0.169	31 ± 9.1	25.8 ± 8.2	0.0008
CD4/CD8 ratio	2.3 ± 1.7	2.9 ± 3	0.176	1.9 ± 0.7	2.5 ± 0.95	0.0002
B-cells	3.7 ± 2.3	2.2 ± 1.4	0.006	10.10 ± 6.2	4.5 ± 2.9	<0.0001
NK cells	5.03 ± 6.3	1.7 ± 1.8	0.001	8.3 ± 13.3	3.7 ± 5.9	<0.0001

**Table 3:** The effect of the hemodialysis session on different subsets of immune cells based on age stratification.

Immune Cells	Pre-Dialysis	Post-Dialysis	*p* Value
21–40 Years Old			
T-cells	10.33 ± 10.72	5.7 ± 3.6	0.626
CD4^+^ T-cells	49.2 ± 5.7	53.8 ± 3	0.626
CD8^+^ T-cells	30.73 ± 5.1	25.8 ± 3.7	0.375
CD4/CD8 ratio	1.6 ± 0.1	2.1 ± 2.6	0.125
B-cells	5.9 ± 5.8	2.7 ± 1.5	0.125
NK cells	2.153 ± 1.85	1 ± 0.39	0.125
41–50 Years Old			
T-cells	16.18 ± 13	6.8 ± 4.9	0.0137
CD4^+^ T-cells	52.5 ± 14.3	55.6 ± 13.8	0.084
CD8^+^ T-cells	31.25 ± 12.6	26.3 ± 11.7	0.004
CD4/CD8 ratio	2.2 ± 1.9	3.1 ± 3.3	0.0020
B-cells	8.7 ± 8.1	3.3 ± 2.6	0.0020
NK cells	3.5 ± 2	1.2 ± 0.93	0.0020
51–60 Years Old			
T-cells	13.2 ± 7.3	7.2 ± 2.9	0.0117
CD4^+^ T-cells	56.04 ± 8.2	58.9 ± 8.7	0.359
CD8^+^ T-cells	27.5 ± 8.3	24.3 ± 5.9	0.250
CD4/CD8 ratio	2.3 ± 0.9	2.6 ± 0.96	0.359
B-cells	6.4 ± 3.6	3.1 ± 1.1	0.0195
NK cells	6.03 ± 5.1	2.34 ± 2	0.0195
>60 Years Old			
T-cells	9.44 ± 2.8	7.8 ± 2.6	0.0625
CD4^+^ T-cells	54 ± 8.3	54.7 ± 9.2	0.8125
CD8^+^ T-cells	30.5 ± 4.8	25.5 ± 5.3	0.187
CD4/CD8 ratio	1.8 ± 0.5	2.2 ± 0.51	0.375
B-cells	7.7 ± 4.4	5.3 ± 4.6	0.0625
NK cells	19 ± 22.1	8.3 ± 9.3	0.0625

## Data Availability

The data that support the findings of this study are available on request from the corresponding author, Waleed Mahallawi.
